# Mechanisms and Advances in Plant Lipid Regulatory Responses Under Biotic and Abiotic Stress

**DOI:** 10.3390/genes17080947

**Published:** 2026-08-13

**Authors:** Xiaohui Pan, Qiufei Wu, Lixia Zhou

**Affiliations:** 1 State Key Laboratory of Tropical Crop Breeding, Chinese Academy of Tropical Agricultural Sciences, Sanya 572025, Chinaqiufei@catas.cn (Q.W.); 2 Coconut Research Institute, Chinese Academy of Tropical Agricultural Sciences, Wenchang 571339, China

**Keywords:** environmental stress, plant lipid metabolism, regulation mechanism, oilseed crops, lipidomics

## Abstract

Biotic stresses (pest feeding, pathogenic fungal/bacterial/viral infection) and diverse abiotic stresses (extreme temperature, drought, waterlogging, saline–alkali soil, heavy metal pollution, nutrient deficiency, UV-B, ozone) severely restrict crop growth and global agricultural yield. Lipids act as core membrane structural constituents and vital secondary signaling messengers, executing multi-layered adaptive balancing functions during cell-type interactive stress acclimation, rather than uniform whole-plant lipid responses. They sustain membrane structural integrity across distinct cell populations, serve as synthetic precursors of bioactive signaling molecules, and trigger cascaded transcriptional and metabolic reprogramming upon environmental stimuli to rebalance physiological status among different cell types. This review systematically summarizes cell-type interactive lipid-mediated plant defense and acclimation balance mechanisms across biotic and abiotic stress contexts. We elaborate the biological functions of fatty acids, phospholipids, galactolipids, sphingolipids and their derivatives (jasmonate, salicylic acid, phosphatidic acid, oxylipin) in stress signal transduction and antioxidant defense and strictly distinguish two categories of lipid changes under all stress types: active adaptive lipid remodeling and passive stress-induced lipid oxidative damage. Key contents include stress-triggered cell-type-specific membrane lipid remodeling, the hierarchical transcriptional regulatory network mediated by WRI1, LEC1, PHR, MADS and other transcription factors governing oil metabolism, as well as crosstalk between lipid metabolism and compartmentalized reactive oxygen species (reactive oxygen species (ROS)) signaling. We further compare conserved lipid-regulatory modules and species-specific divergent responses across model plants and economic oilseed crops, integrating state-of-the-art targeted/untargeted lipidomics, single-cell spatial lipidomics and multi-omics joint breeding strategies to improve multi-stress tolerance in oilseed crops. By consolidating global research progress up to 2025, including the two latest 2026 cross-species meta-analysis reviews, this review provides systematic theoretical support and operable multi-level technical frameworks for genetic engineering targeting conserved lipid pathways to breed stress-resilient high-oil crop germplasm, and highlights reliable lipid stress biomarker screening as a promising translational research direction.

## 1. Introduction

**Figure 1:**
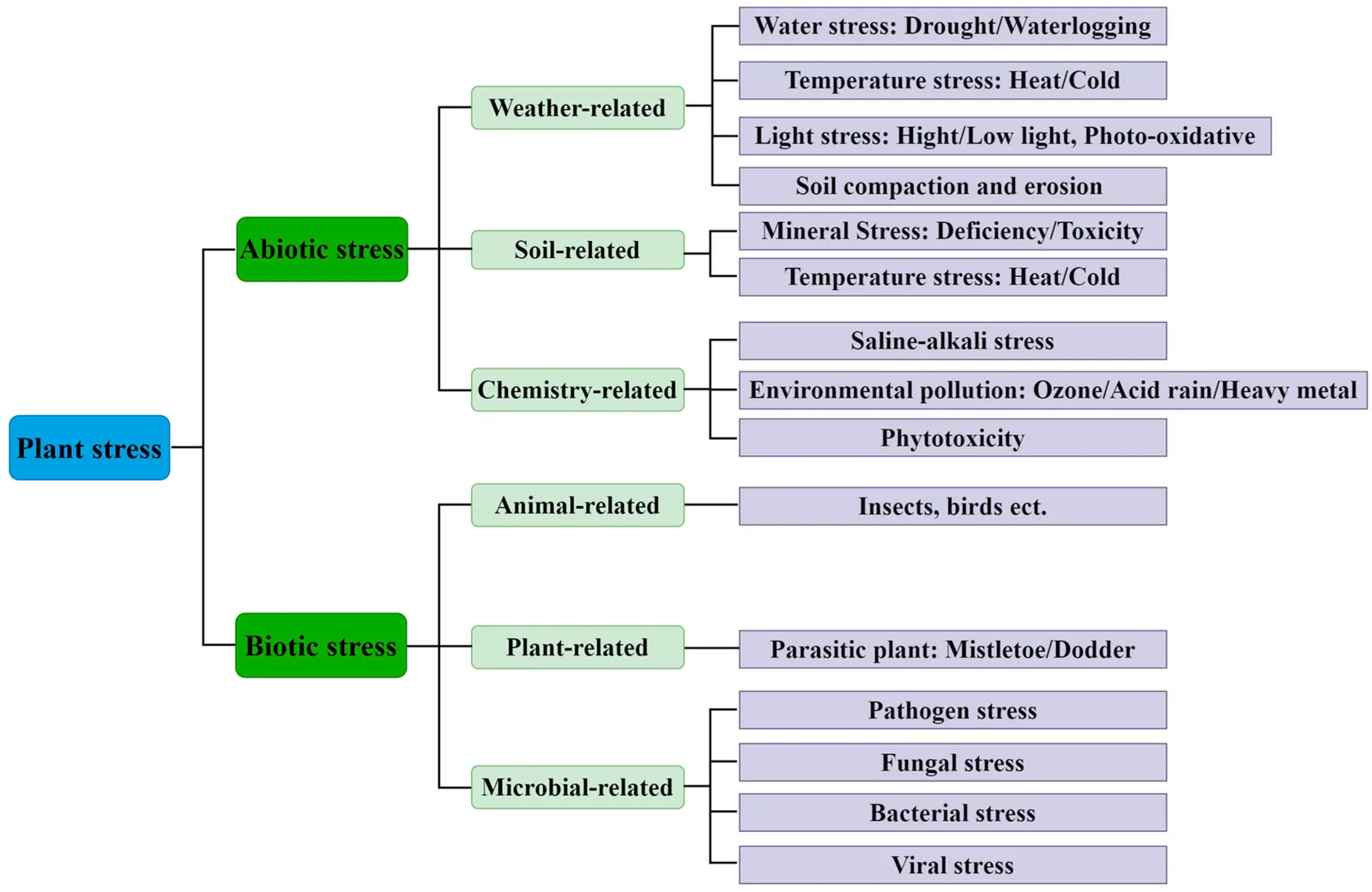
Classification of plant biotic and abiotic environmental stresses. Note: Microbial-related biotic stress only refers to pathogenic microorganism infection (pathogenic fungi, bacteria, viruses); beneficial symbiotic microbes including arbuscular mycorrhizal fungi and endophytic bacteria are excluded from this stress category, which can improve host stress tolerance via regulating plant lipid metabolism. Salt-alkali stress is uniformly classified as a soil-related abiotic stress.

Plant stress refers to adverse environmental factors that disrupt the balanced growth, development and yield performance of plant cell populations, and it is generally divided into biotic stress and abiotic stress (Figure 1) [1,2,3]. Mild stress activates genetically programmed intrinsic adaptive physiological responses among different cell types to restore intercellular metabolic balance, while severe stress causes irreversible lipid oxidative damage, developmental defects and massive yield losses, limiting the geographic cultivation range of crops [4]. Lipids are classified into two major membrane lipid subfamilies (phospholipids, glycolipids) and neutral storage lipids (triacylglycerol, TAG), including phospholipids, galactolipids, sphingolipids and TAGs [5]. As the primary building blocks of diverse cell-type membrane systems, lipids participate in energy storage, carbon redistribution and intercellular signal transduction to coordinate multi-stress adaptive balance across mesophyll, guard, root cortical and pericycle cells [5]. TAGs, collectively referred to as seed storage oils, function as critical carbon reservoirs to maintain plant survival under nutrient deprivation and oxidative damage in both vegetative tissues and developing seeds [6]. Beyond structural roles, multiple lipid species serve as core intercellular signaling messengers with cell-type-specific response patterns. The phosphatidylinositol (PI) signaling pathway generates inositol trisphosphate (IP_3_) and diacylglycerol (DAG) to mobilize intracellular Ca^2+^ and activate downstream cascades under osmotic and oxidative stress, with distinct signal intensity in epidermal and mesophyll cells [7,8]. Sphingolipids (long-chain bases, ceramides) modulate programmed cell death (PCD) and abscisic acid (ABA) signal transduction in root and leaf cell populations [9]. Galactolipid turnover in chloroplasts of mesophyll cells supplies precursors for jasmonic acid (JA) and oxylipin biosynthesis to mediate systemic defense signal transport to adjacent cell types [10]. Phosphatidic acid (PA), produced via phospholipase D (PLD) or phospholipase C-diacylglycerol kinase (PLC-DGK) cascades, accumulates rapidly under salt, drought and cold stress across all root cell layers; PA interacts with target proteins such as ABI1 and CTR1 to modulate cell-type-differentiated stress responses [11,12]. Collectively, these lipid branches constitute an integrated intercellular signal transduction axis connecting environmental perception in specialized sensor cells (guard cells, root epidermis) to whole-plant physiological reprogramming.

### 1.1. Cell-Type Heterogeneity of Plant Lipid Remodeling: The Interactive Balance Model

A critical limitation of previous stress lipid research lies in the over-simplified “average whole-plant model”, ignoring the fact that plant organs are composed of functionally differentiated cell types with independent developmental trajectories, epigenomes, and transcriptomes and unique lipidomes [13]. Identical external stress triggers divergent lipid remodeling patterns among distinct cell populations, and ultimate plant stress adaptation is essentially a dynamic rebalancing of lipid metabolism and signal transduction between heterogeneous cell types, rather than uniform lipid changes across the entire tissue. For example, guard cells act as primary temperature and drought sensors without peroxisomes, relying on plasma membrane phospholipid remodeling and rapid PA signal accumulation to regulate stomatal movement; mesophyll cells contain abundant chloroplasts and peroxisomes, prioritizing galactolipid desaturation to stabilize photosynthetic membranes under thermal and osmotic stress; root cortical and pericycle cells show cell-layer-specific TAG storage accumulation under flooding hypoxia to buffer energy deficit [13,14]. Single-cell lipidomics technology has recently validated that localized ROS bursts and lipid peroxidation only occur in specific root cell layers under saline-alkali and flooding stress, while neighboring cell populations activate adaptive lipid remodeling to offset injury signals via intercellular lipid messenger transport. This cell-type interactive balance perspective runs through all mechanistic discussions in this review, replacing the traditional average tissue model to interpret lipid-mediated stress tolerance.

### 1.2. Lipid–Hormone Crosstalk and Lipid Remodeling Under Nutrient Deficiency

Nutrient stress not only includes single macro/microelement deficiency (phosphorus (P), nitrogen (N), iron (Fe), zinc, and potassium), but also multi-nutrient ratio imbalance and soil redox disorder-induced indirect ion bioavailability limitation, representing a major category of abiotic stress triggering genome-wide cell-type-differentiated lipid remodeling as an evolutionarily conserved adaptive strategy across higher plants [15,16,17]. Under phosphate-deficient conditions, plants globally replace membrane phospholipids with galactolipids and sulfoquinovosyldiacylglycerol (SQDG) across root and leaf cell populations to remobilize inorganic phosphate for essential metabolic reactions. The transcription factor PHOSPHATE STARVATION RESPONSE 1 (PHR1) acts as a core molecular switch to upregulate *NPC4* and *GCS* expression, which further drives the conversion of sphingophospholipids to sphingoglycolipids and maintains membrane integrity under low-phosphate stress in root epidermal cells [15,18].

Apart from single phosphorus limitation, nitrogen starvation, iron deficiency and multi-nutrient co-limitation trigger distinct cell-type-specific lipid metabolic reprogramming. Specifically, nitrogen deprivation leads to massive triacylglycerol (TAG) accumulation in root cortical cell lipid droplets and comprehensive membrane lipid turnover across leaf mesophyll cells; such lipid catabolism provides recyclable carbon skeletons and energy reserves to sustain slow plant growth under nitrogen-limited circumstances [19,20]. Differently, iron deficiency severely represses the transcription of polyunsaturated fatty acids in thylakoid galactolipids, disrupts chloroplast structural stability, and eventually impairs photosynthetic carbon fixation [21,22]. Soil low redox potential reduces soluble iron and manganese availability, inducing indirect nutrient deficiency stress and further amplifying lipid peroxidation in root cell membranes.

Beyond independent lipid reprogramming responding to individual nutrient stresses, sophisticated crosstalk between lipid metabolism and phytohormone signaling is indispensable for coordinating root morphological plasticity and photosynthetic homeostasis under combined nutrient and environmental stresses, with endogenous hormone distribution across root cell layers shaping divergent lipid remodeling responses [23,24,25]. As a typical lipid-derived hormone, jasmonate (JA) is synthesized from plastidial α-linolenic acid in mesophyll cells and governs plant defense against herbivores and necrotrophic pathogens via intercellular signal transport [26,27]. Meanwhile, abscisic acid (ABA) signaling can rapidly activate phospholipase D (PLD) activity in guard cells upon osmotic stress, which subsequently produces phosphatidic acid (PA), a vital lipid second messenger, to induce stomatal closure [28,29]. These examples underscore the central role of lipid-derived signaling molecules in integrating hormone gradient pathways during cell-type balanced stress adaptation [30].

Multiple lipid molecules serve as key intercellular signal mediators to bridge nutrient stress responses and phytohormone regulatory cascades. For instance, digalactosyldiacylglycerol (DGDG) synthesized in mesophyll chloroplasts integrates phosphate starvation signals into JA biosynthesis pathways, as evidenced by the finding that *OsDGD1* knockout in rice increases the MGDG:DGDG ratio and enhances endogenous JA levels and systemic signaling transmission to root tissues [31]. Meanwhile, the DGK-PA signaling module dynamically modulates ABA-dependent stress responses across leaf cell populations. Mechanistically, DGK5 and its lipid product phosphatidic acid (PA) physically interact with ABA2, a key ABA biosynthesis enzyme, to suppress its enzymatic activity and promote its nuclear sequestration in mesophyll cells, thereby fine-tuning ABA production and downstream ABA signaling outputs in guard cells [32]. In addition, overall plant nutrient status reshapes the balance between JA and salicylic acid (SA) signaling pathways across organ cell populations, thereby modifying plant susceptibility to pathogenic infection. In summary, these interactive pathways constitute a multilayered regulatory network that links cell-type-differentiated lipid metabolic remodeling, mineral nutrient adaptive balance and plant immune responses.

### 1.3. Research Gaps and Review Framework

Despite considerable progress in plant lipid stress biology over recent decades, several critical knowledge gaps persist that constrain both mechanistic understanding and the translational application of lipid regulatory networks in stress adaptation, which this review systematically addresses with updated 2020–2026 literature including the two latest cross-species meta-analysis reviews [14,33]. First, existing studies have disproportionately focused on a limited set of model crops, particularly *Arabidopsis thaliana*, whereas systematic comparative investigations across diverse economic oilseed and cereal crops (maize, wheat, rice, rapeseed, sunflower) remain insufficiently integrated. This taxonomic narrowness limits the generalizability of current lipid stress paradigms and impedes the transfer of core conserved mechanisms into broadly applicable crop improvement strategies; the distinction between model-specific non-transferable pathways and cross-species conserved lipid regulatory modules requires explicit sorting. Second, most reviews published before 2020 lack comprehensive coverage of phosphoinositide (PI)- and sphingolipid-mediated cell-type-differentiated signaling cascades, which have recently emerged as central hubs in plant stress signal transduction [33,34]. Phosphoinositides comprise seven distinct isoforms, including PtdIns3P, PtdIns4P, PtdIns5P, and their bis- and trisphosphate derivatives, functioning as key precursors for inositol phosphates and diacylglycerol and extensively involved in membrane trafficking and cell-type-specific stress-responsive processes. As major plasma membrane constituents, sphingolipids account for up to 40% of plant epidermal cell membrane lipids, playing essential roles in programmed cell death and lipid raft microdomain formation [35]. Nevertheless, earlier reviews have rarely addressed the mechanistic links between these signaling lipid classes and plant responses to multi-nutrient imbalance stress, with cell-type heterogeneity completely ignored [35]. Third, previous reviews have largely neglected three emerging and functionally significant frontiers in plant lipid stress biology. These include (1) the interplay between lipid metabolism and non-specific lipid transfer proteins (nsLTPs), which mediate intercellular fatty acid transport and serve dual functions in pathogen defense and stress modulation [36]; (2) the dynamic turnover of cell-type-specific lipid droplets (LDs) and the regulatory role of LD-plasma membrane contact sites in maintaining cellular lipid homeostasis under stress conditions, although mechanistic insights remain limited [37,38]; and (3) single-cell spatial lipid heterogeneity, which has long remained intractable owing to technical constraints and now constitutes a pivotal frontier for deciphering intercellular adaptive coordination [39]. Fourth, a critical shortcoming of prior reviews is their failure to rigorously distinguish between two fundamentally distinct lipid phenotypic responses that occur across all stress conditions, thereby obscuring meaningful biological interpretation. One response is adaptive lipid remodeling, a genetically orchestrated, reversible process that bolsters stress tolerance through regulated fatty acid desaturation, phospholipid-to-galactolipid substitution, controlled generation of lipid second messengers, and stress-inducible triacylglycerol (TAG) accumulation for energy buffering. The other is stress-induced lipid damage, a passive and irreversible degradative outcome triggered by excessive reactive oxygen species (ROS) bursts that overwhelm endogenous antioxidant systems, characterized principally by unchecked lipid peroxidation and indiscriminate loss of polyunsaturated fatty acids. This damage represents a pathological consequence of severe stress rather than an adaptive protective mechanism.

### 1.4. Conserved Core Lipid Regulatory Modules Across All Biotic and Abiotic Stress

To resolve the fragmented analytical limitation caused by separating each stress type for independent discussion in previous research, this section systematically summarizes four evolutionarily conserved lipid functional modules shared by nearly all biotic and abiotic stress response processes, which constitute the universal core adaptive framework of plant lipid metabolism under adverse environments. The first module refers to the membrane lipid remodeling regulatory system: facing temperature fluctuation, drought, salinity and pathogenic infection, all plant cell populations will initiatively adjust the ratio of saturated and unsaturated fatty acids, as well as the composition of glycerolipids such as phospholipids and galactolipids, so as to dynamically maintain membrane fluidity, physical barrier integrity and selective ion permeability, which is the most basic lipid adaptive strategy widely conserved across angiosperms [34]. The second module centers on lipid second messenger signal cascades: phosphatidic acid (PA), sphingolipid long-chain bases and multiple oxylipins function as diffusible intercellular signaling substances, transferring stress perception signals from specialized sensor cells (guard cells, root epidermal cells) to whole tissues, thereby triggering genome-wide transcriptional reprogramming downstream of lipid signal transduction [40]. The third module mediates lipid–hormone interactive crosstalk: jasmonic acid (JA), salicylic acid (SA) and abscisic acid (ABA) are all synthesized relying on lipid precursors, and their concentration gradients among different cell populations are mutually regulated by lipid metabolic flux, coordinating the balance between plant immune response and osmotic stress adaptation at the tissue and organ levels [41]. The fourth module forms a bidirectional lipid–ROS feedback regulatory loop: moderate reactive oxygen species (ROS) accumulation induced by mild stress will trigger regulated fatty acid desaturation and antioxidant lipid remodeling to clear redundant oxidants, while sustained excessive ROS bursts under severe stress will irreversibly induce membrane lipid peroxidation and irreversible cellular injury; the threshold of ROS concentration determines whether lipid remodeling belongs to active adaptation or passive damage, forming a binary regulatory switch controlling the plant survival state.

Based on the above four cross-stress conserved lipid modules, this review constructs a multi-dimensional analytical framework centered on cell-type interactive balance and proposes four major innovative improvements compared with previously published review studies to fill existing research gaps. First, this paper integrates a large volume of lipidomics and single-cell multi-omics research data published after 2020; systematically sorts out the conserved lipid regulatory pathways shared by model plants and economic crops, as well as species- and cell-type-specific divergent remodeling patterns; and strictly distinguishes adaptive lipid reprogramming from stress-triggered lipid oxidative damage in all chapters to eliminate ambiguity in discussions on the biological significance of lipid phenotypic changes [42]. Second, the present work supplements a systematic collation of plant lipid oxidation and signal responses under UV-B and ozone stress, two environmental stress categories rarely comprehensively summarized in previous review articles, enriching the coverage of abiotic stress lipid-regulatory mechanisms. Third, we consolidate the hierarchical transcriptional regulatory networks governing the whole lipid biosynthesis pathway, with focused discussion on three core transcription factor families, namely PHR, MADS-box and WRI1, and clarify their cell-type-differentiated target gene activation modes during stress-induced oil metabolism reprogramming. Fourth, this review puts forward operable multi-level technical routes integrating conventional bulk lipidomics, single-cell spatial lipidomics and multi-omics joint breeding, establishes a bridge connecting basic cell lipid biology and agricultural practical improvement, and provides targeted translational theoretical guidance for the genetic engineering breeding of multi-stress-resistant oilseed germplasm [40].

## 2. Lipid Response to Biotic Stress

Biotic stress mainly includes herbivorous insect feeding and pathogenic microorganism infection (fungi, bacteria, viruses), which disrupt intercellular lipid balance and trigger cell-type-differentiated lipid defense remodeling or lipid peroxidation damage. Lipids function as dual regulators: physical barrier structural components and mobile intercellular signal messengers coordinating whole-tissue systemic immunity.

### 2.1. Insect Herbivory

Insect infestation represents a major biotic factor limiting global crop yield. As sessile organisms, plants cannot escape insect attacks via physical movement and have evolved cell-type-specific biochemical defense systems to adjust lipid composition and specialized metabolite production in epidermal and mesophyll cells in response to herbivory [28]. Lipids participate in plant anti-insect defense through two core functional modes: fatty acids and their degradation derivatives exhibit direct insecticidal activity, while epidermal phospholipids and cuticular waxes form pre-existing physical and chemical barriers to reduce insect feeding and colonization [43].

#### 2.1.1. Insecticidal Effects of Fatty Acids and Their Derivatives

Fatty acids (FAs), as fundamental constituents of membrane phospholipids synthesized in plastids of mesophyll cells, are pivotal intercellular mediators of plant defense responses [44,45]. When plants are subjected to insect pest attacks, wound signals trigger rapid accumulation of long-chain FAs and FA degradation products in damaged epidermal cells, which are transported to adjacent undamaged cell populations to enhance whole-leaf immune functions [46]. Carbon chain length determines FA insecticidal activity: C18 fatty acids (oleic acid C18:1, linoleic acid C18:2, α-linolenic acid C18:3) significantly improve plant resistance to piercing–sucking and chewing pests [47]. Specifically, increased plastidial C18:1 content enhances plant broad-spectrum pest and disease resistance. In *Arabidopsis*, plastid C18:1 homeostasis is regulated by glycerol 3-phosphate (G3P) acylation in the fatty acid synthesis pathway, and the G3P-C18:1 balance controls the equilibrium between SA and JA intercellular signaling gradients, which are essential for coordinating cell-type balanced plant defense responses across epidermal and mesophyll tissues [47].

Beyond primary fatty acid metabolites, plants synthesize lipid-derived secondary metabolites to repel, trap or kill pests, which can be extracted as eco-friendly botanical pesticide raw materials supporting integrated pest management (IPM) strategies [48,49].

Fatty acid desaturase (FAD) gene family members localized in plastids and the endoplasmic reticulum catalyze saturated fatty acid desaturation to generate polyunsaturated FAs with pest-resistance functions, showing species-specific divergent expression patterns under insect feeding [50]. For example, tomato *SlFAD7* overexpression elevates linoleic acid and hexadecenoic acid accumulation in leaf epidermal cells, significantly improving aphid resistance, while *SlFAD2* regulates mesophyll cell FA composition to modulate plant susceptibility to aphid infestation [51]. These findings highlight cell-type-differentiated fatty acid desaturation as a core adaptive lipid remodeling strategy against insect biotic stress, rather than passive lipid damage.

#### 2.1.2. The Insecticidal Role of Phospholipids and Cuticular Waxes

Epidermal cell surface lipid layers and cuticular waxes are constitutive structural lipids forming physical pre-formed barriers to reduce insect landing, feeding and oviposition colonization [52]. JA signaling synthesized from mesophyll α-linolenic acid acts as the central regulatory hub governing plant anti-insect systemic defense: insect feeding and mechanical wounds trigger rapid JA biosynthesis in damaged cells; JA and methyl jasmonate (MeJA) interconvert to realize long-distance intercellular and inter-organ defense signal transmission, upregulating defense gene expression and defensive lipid metabolite accumulation in undamaged tissues to restrict insect expansion [53,54].

Aliphatic compounds dominate plant surface wax composition in epidermal cells, with fatty acid carbon chain length differentially regulating aphid behavioral responses: short-chain FAs (C8–C13, e.g., dodecanoic acid C12) inhibit winged peach aphid landing and settlement; long-chain FAs above C16 conversely attract aphid colonization, forming a cell-surface lipid signal gradient mediating plant-insect interaction [55,56]. Herbivore-induced plant volatiles (HIPVs), synthesized via JA-regulated lipid metabolic pathways in mesophyll cells, are critical indirect defense bioactive compounds: HIPVs released from wounded tissues repel continuous herbivory, attract pest natural enemies, and mediate intra- and inter-plant defense signal communication to establish whole-plant systemic resistance [57,58]. In tea plants, mild pre-harvest insect feeding triggers adaptive lipid remodeling in leaf mesophyll cells, activating secondary metabolic reprogramming and increasing polyphenol and aromatic lipid-derived volatile accumulation, simultaneously improving tea flavor quality and pest resistance, representing a typical beneficial stress-induced lipid metabolic modification without irreversible lipid damage [59].

### 2.2. Pathogen Infection

Plant pathogens mainly include pathogenic fungi, bacteria, mycoplasmas and viruses, which break intercellular lipid balance and induce two opposite lipid phenotypes—adaptive immune lipid remodeling in resistant varieties or severe lipid peroxidation damage in susceptible varieties—causing annual global crop yield loss of approximately 15% [60]. Lipids mediate plant immunity via three pathways: epidermal cuticle lipid physical barrier formation, synthesis of immune signaling precursors, and modulation of cell-type balanced SA-JA immune gradient crosstalk [61].

#### 2.2.1. Antibacterial and Antifungal Effects of Fatty Acids and Their Derivatives

The aerial epidermal cell surface is covered by a highly hydrophobic fatty-acid-derived cuticle layer, forming a primary physical barrier blocking pathogen penetration and tissue colonization [62]. Polyunsaturated C18 fatty acids (linoleic acid C18:2, α-linolenic acid C18:3) accumulated in mesophyll cells show direct antifungal activity against necrotrophic pathogens: soybean varieties with high C18:2/C18:3 content exhibit strong resistance to Botrytis cinerea gray mold, while varieties with reduced polyunsaturated FA levels suffer severe lipid peroxidation damage and complete loss of gray mold resistance after infection [63].

**Figure 2:**
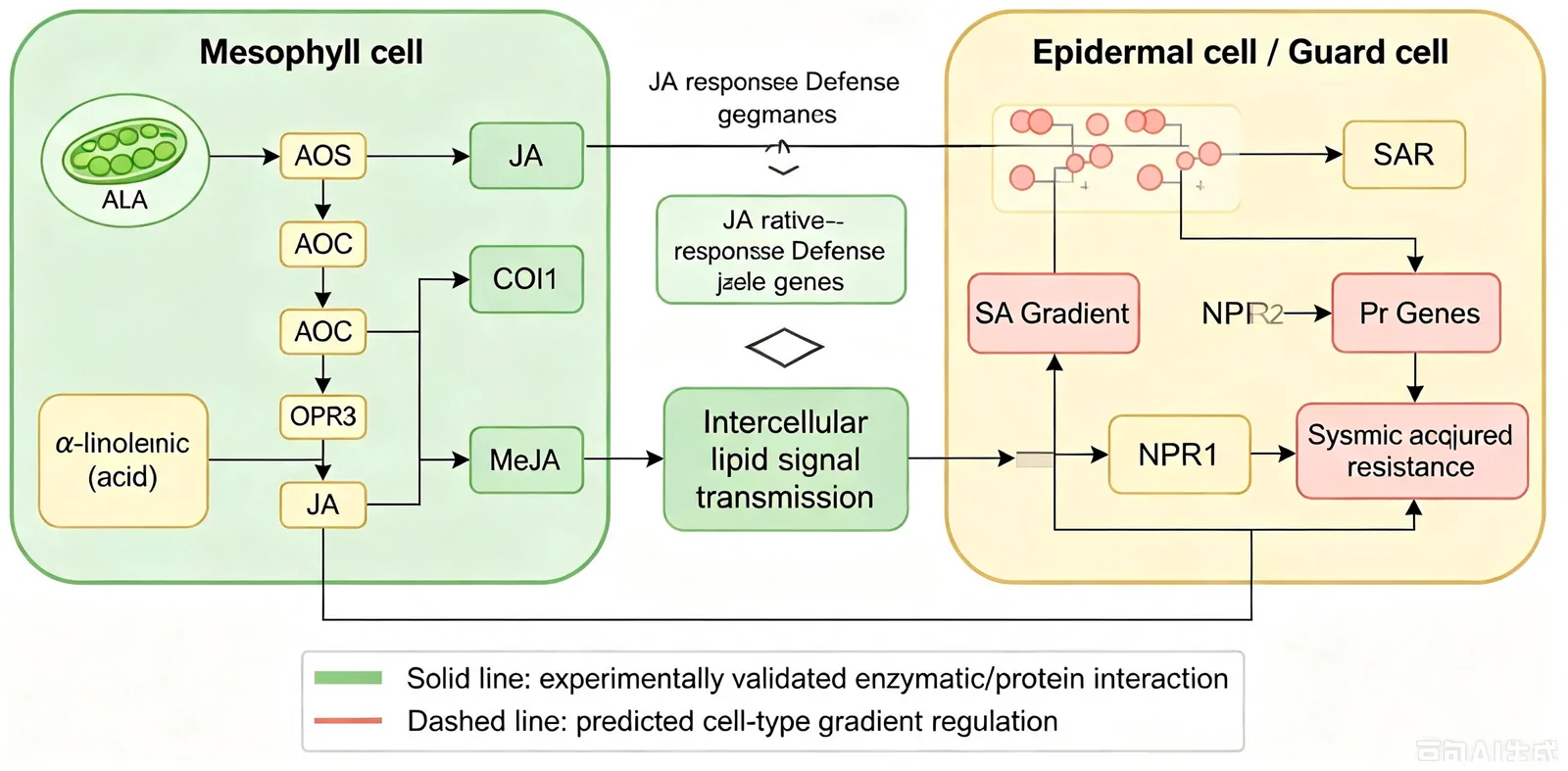
Regulatory crosstalk between salicylic acid (SA) and jasmonic acid (JA) in plant immunity. ALA: α-linolenic acid, JA: jasmonic acid, COI1: jasmonate receptor protein, JAZ: jasmonate Zim domain protein, SA: salicylic acid, HR: hypersensitive response cell death, SAR: systemic acquired resistance. Note: SA acts as a key regulator balancing immune responses among different cell types within single organs and across distant tissues, coordinating intercellular defense signal gradients beyond only PR gene induction. Solid lines: experimentally validated regulatory interactions, dashed lines: putative predictive regulatory models requiring further functional verification [7].

JA and SA are two core lipid-derived immune hormones with cell-type-differentiated antagonistic and synergistic crosstalk patterns (Figure 2) [64]. JA biosynthesis initiates from plastidial α-linolenic acid (18:3 ALA) in mesophyll cells, sequentially catalyzed by lipoxygenase, allene oxide synthase (AOS), allene oxide cyclase (AOC), and OPR3 [65]. JA gradient distribution across leaf cell populations mainly enhances plant resistance to necrotrophic fungi by inhibiting excessive programmed cell death and activating epidermal defense responses. In contrast, SA signaling dominates mesophyll cell defense against biotrophic pathogens, triggering local acquired resistance (LAR) and systemic acquired resistance (SAR) via intercellular signal transport [66]. SA modulates antioxidant enzyme activity gradients among cell types: SA promotes superoxide dismutase (SOD)-mediated moderate ROS generation in mesophyll cells while inhibiting catalase (CAT) and ascorbate peroxidase (APX) activity in epidermal cells, forming a cell-balanced ROS signal gradient to amplify immune responses without triggering large-scale lipid peroxidation damage [67,68]. Exogenous glycerol treatment improves wheat powdery mildew resistance by activating root–leaf intercellular lipid signal transport, altering G3P and C18:1 metabolism gradients across leaf cell populations; reduced epidermal C18:1 accumulation correlates with enhanced powdery mildew resistance, indicating C18:1 negatively regulates epidermal cell immune responses against this fungal disease [69,70].

#### 2.2.2. Regulation of Phospholipids in Resistance to Pathogen Infection

Phospholipases (PLD, PLC) are core enzymes driving cell-type-differentiated membrane lipid remodeling under pathogen infection, with resistant plant varieties maintaining sustained high phospholipase activity in mesophyll and epidermal cells to trigger controlled PA signal accumulation (adaptive remodeling), while susceptible varieties show rapid phospholipid degradation and uncontrolled lipid peroxidation damage after infection [71]. Phosphatidic acid (PA), generated via DGK catalysis in diverse cell populations, functions as both a lipid biosynthesis intermediate and universal immune second messenger [72]. Pathogen infection significantly upregulates DGK gene expression in tomato, rice, tobacco and Arabidopsis mesophyll cells; DGK-derived PA modulates plasma membrane surface charge and fluidity, promoting defense protein aggregation and activation at epidermal cell membrane lipid rafts to restrict pathogen colonization [73]. Coordinated activation of phospholipases and DGK triggers rapid PA accumulation in infected cell populations, releasing free fatty acids, JA and PA intercellular lipid signals to synergistically activate whole-tissue immune responses and inhibit pathogen proliferation without inducing broad cell death [74]. All PA-mediated SA/JA crosstalk regulatory relationships listed in Table 1 below are classified by experimental evidence level.

## 3. Lipid Response to Abiotic Stress

Abiotic stress covers extreme temperature, drought, waterlogging, saline–alkali soil, heavy metal pollution, UV-B and ozone, all triggering cell-type-differentiated lipid adaptive remodeling or oxidative damage depending on stress intensity and cell physiological characteristics. Lipids maintain membrane stability via fatty acid composition adjustment and coordinate compartmentalized ROS signal balance across distinct cell populations.

### 3.1. Temperature Stress: Lipid Remodeling Mediates Cell-Type Thermo-Balance

**Figure 3:**
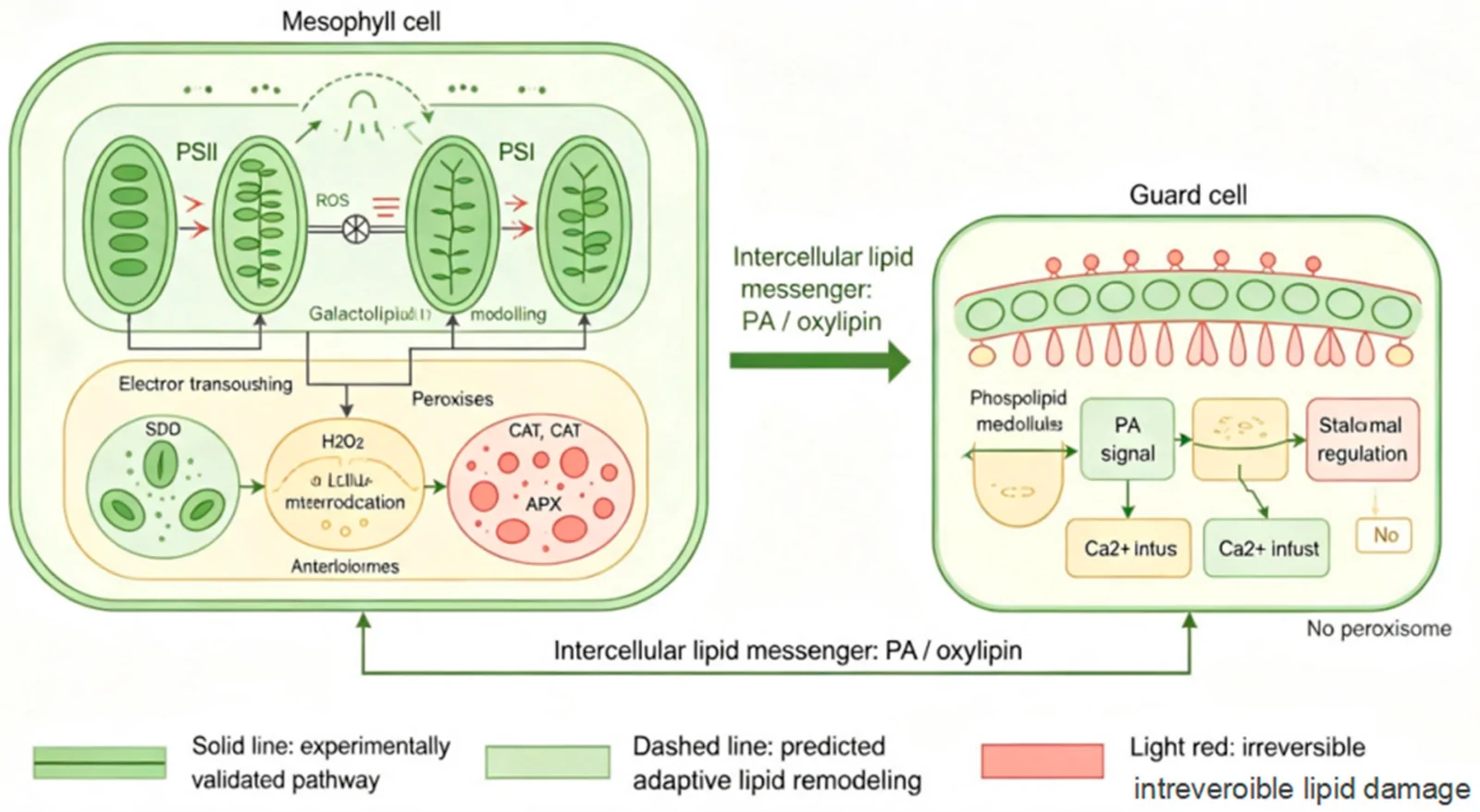
Accumulation and clearance of compartmentalized ROS under temperature stress. PSII: photosystem II; PSI: photosystem I; ROS: reactive oxygen species. Note: Chloroplast PSI/PSII electron transport chain ROS production exclusively occurs in leaf mesophyll cells with complete thylakoid membranes; guard cells act as primary temperature sensor cells but contain no peroxisomes, so peroxisomal ROS metabolism only functions in mesophyll cell populations to jointly coordinate whole-leaf temperature stress adaptive balance via intercellular lipid signal transport. Solid lines = validated direct regulatory pathways; dashed lines = indirect multi-step metabolic crosstalk. Solid arrows indicate activation, and dashed arrows indicate inhibition.

Lipids mediate plant temperature responses by adjusting membrane fluidity across mesophyll, guard and root cell populations, balancing structural stability and intercellular signal transduction under thermal fluctuation [75]. Under low temperature, mesophyll and root cells activate fatty acid desaturation adaptive remodeling to increase polyunsaturated FA proportion and counteract membrane rigidification; under high temperature, cell-type-specific saturated FA accumulation stabilizes membrane structure to suppress excessive fluidity and thermal denaturation of membrane proteins [76]. Beyond structural adjustment, lipid-derived PA and JA act as intercellular second messengers to activate temperature stress transcription factor cascades across tissue layers, coordinating antioxidant system activation and stress-tolerance gene expression [77]. The balance between adaptive fatty acid remodeling and temperature-induced lipid peroxidation damage determines plant thermotolerance (Figure 3).

#### 3.1.1. High Temperature Stress

Global climate warming aggravates high-temperature stress, with 3–4 °C average temperature rise predicted to trigger 15–40% regional crop yield loss across Asia and Africa [78,79]. High temperatures severely suppress oilseed crop seed TAG storage accumulation during maturation and disrupt mesophyll photosynthetic membrane function in vegetative tissues, representing dual damage to vegetative stress adaptation and reproductive yield formation [80]. Heat stress breaks membrane lipid balance in mesophyll cells: elevated plasma membrane permeability, reduced polyunsaturated fatty acid proportion, and ROS burst-induced lipid peroxidation damage under severe heat; mild heat triggers adaptive saturated FA enrichment to stabilize thylakoid membranes [81]. Endogenous SA and ABA gradient distribution across cell populations coordinate antioxidant defense: SA enhances mesophyll cell antioxidant enzyme activity to alleviate heat-induced lipid peroxidation, while ABA regulates guard cell PA signal accumulation to close stomata and reduce transpirational water loss under combined heat-drought stress [82]. Heat stress represses fatty acid desaturase gene transcription in mesophyll chloroplasts, reducing polyunsaturated galactolipid content and decreasing membrane fluidity, ultimately weakening thermotolerance if adaptive remodeling cannot offset lipid damage [83]. Transcription factors including GmABI3 in soybean coordinate heat stress lipid remodeling in both leaf vegetative cells and developing seed storage cells: heterologous overexpression in Arabidopsis increases seed TAG content by 7.3% and modulates fatty acid composition to enhance heat resilience of both vegetative and reproductive tissues [84].

#### 3.1.2. Low-Temperature Stress

Low-temperature stress induces two distinct lipid phenotypes based on intensity: above-freezing mild cold activates adaptive fatty acid desaturation remodeling in root and mesophyll cells to increase polyunsaturated FA proportion and maintain membrane fluidity; freezing severe cold causes irreversible plasma membrane lipid peroxidation damage and cell leakage [85,86]. Key lipid biosynthetic enzymes GPAT and FAD show cell-type-differentiated cold-induced upregulation: plastid GPAT overexpression in mesophyll cells simultaneously enhances cold tolerance and seed oil accumulation, with significant PlGPAT expression elevation in peony mesophyll cells under 4 °C cold stress [87]. Low temperature induces FAD gene transcription to convert linoleic acid to α-linolenic acid (ALA), a JA biosynthesis precursor, and JA intercellular signal gradients accelerate saturated-to-unsaturated FA conversion across cell populations to strengthen cold resistance [88]. Perilla leaf lipidomics under 4 °C cold treatment verify this cell-balanced remodeling mechanism: mesophyll cell linolenic acid peaks at 9 h cold treatment, while exogenous MeJA signal transport advances this peak to 6 h, significantly elevating whole-leaf polyunsaturated FA proportion and cold tolerance [89]. Table 1 summarizes PA, DAG and oxylipin lipid signal modules integrating cold-triggered SA/JA intercellular gradient balance across leaf cell populations.

**Table 1:** Lipid signaling molecules mediating SA/JA cell-type crosstalk, classified by evidence level.

Lipid Signaling Molecules	Core Genes/Enzymes Function	Effects on the SA/JA Pathways	Evidence Level	References
Phosphatidic acid (PA)	Phospholipase D (PLD)	Dual central hub messenger: promotes moderate ROS production in mesophyll cells to activate SA signaling; upregulates JA biosynthetic AOS enzyme in plastids to amplify JA gradients in epidermal cells, coordinating balanced biotic stress defense.	Fully genetically validated (knockout/overexpression + Co-IP protein interaction)	[90]
Diacylglycerol (DAG)	Phospholipase C (PLC)	PLC hydrolyzes membrane glycerolipids to generate DAG and IP_3_; DAG phosphorylated by DGK produces PA, linking PI signaling to SA/JA crosstalk cascade.	Partially validated (lipid quantification + inhibitor treatment, lacking gene knockout)	[91]
Free fatty acid(s) (FFA)	Acyl hydrolase	Saturated FFAs (palmitic acid) act as damage-associated molecular patterns in wounded epidermal cells, directly inducing JA-responsive defense gene expression independent of JA biosynthesis precursors.	Correlative multi-omics evidence only, predictive model without functional verification	[92]
Oxylipins (excluding JA)	LOX, α-DOX	C6 volatile oxylipins and traumatin transmit intercellular wound signals, reshaping SA/JA equilibrium across leaf cell populations to adjust the pathogen resistance spectrum.	Transcriptome-lipidome correlation, putative regulatory model	[93]

### 3.2. Water Stress: Drought and Flooding Trigger Opposite Cell Lipid Remodeling Patterns

#### 3.2.1. Drought Stress

**Figure 4:**
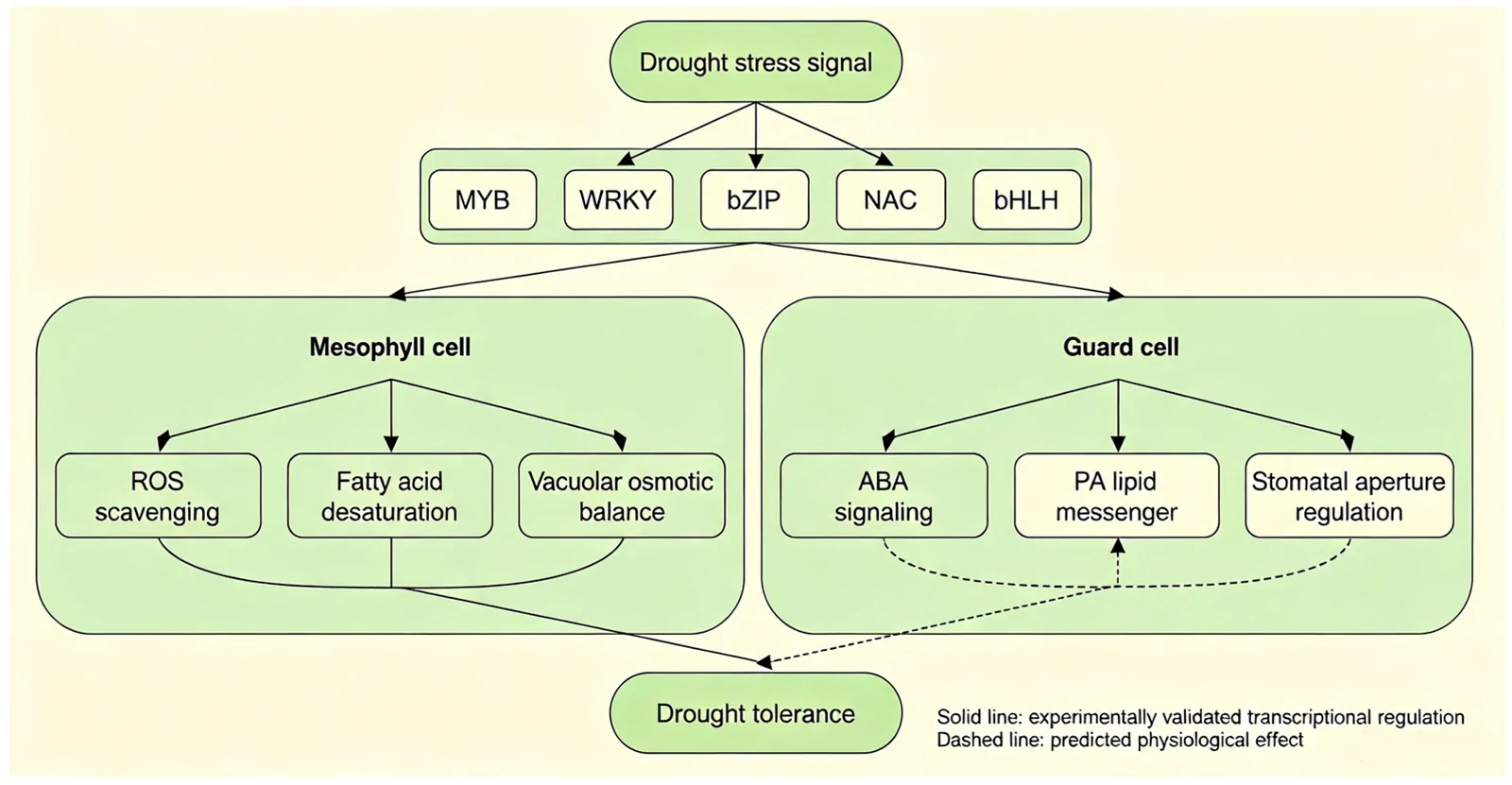
Schematic model of transcriptional-factor-coordinated lipid metabolism remodeling under drought stress. Key TFs (MYB, WRKY, bZIP, NAC, bHLH) show cell-type-differentiated functions: MYB/bZIP mainly drive ROS scavenging in large-vacuole mesophyll cells, and WRKY/NAC/bHLH primarily regulate guard cell pore size adjustment and root cell osmotic balance. Solid lines = experimentally verified direct transcriptional regulation; dashed lines = putative indirect metabolic crosstalk. Revised labels: “ROS scavenging” (original ambiguous “Clear ROS”); “Cell turgor and osmotic pressure balance” (original “Osmotic adjustment”).

Membranes of leaf mesophyll cells, guard cells, and root epidermal and cortical cells show distinct structural stability and lipid remodeling sensitivity under drought stress, due to divergent cell volume, membrane tension and basal lipid composition [9]. Severe drought disrupts cellular ROS scavenging balance in mesophyll cells, triggering excessive ROS bursts, polyunsaturated fatty acid peroxidative degradation and irreversible membrane lipid damage, reducing oil quality in maturing oilseed seeds [94,95]. Mild drought activates adaptive lipid remodeling: moderate saturated FA accumulation stabilizes vacuolar and plasma membranes and enhances antioxidant enzyme activity to scavenge ROS, maintaining cell osmotic homeostasis and drought resilience [96]. Oilseed crops show species-specific divergent seed storage lipid responses during maturation under drought: sunflower seed oil concentration remains stable; rapeseed increases seed protein content while reducing TAG accumulation; sesame suffers simultaneous decline in seed oil and protein content [97,98,99]. Multiple transcription factor families coordinate cell-type-differentiated lipid remodeling under drought (Figure 4): *ZmLEC1* specifically enhances seed TAG synthesis in maize developing embryos; MYB transcription factors function via ABA-dependent mesophyll cell antioxidant regulation or ABA-independent root lipid remodeling (*OsMYB6*); overexpression of *BnLEA3* and *VOC* genes in *Brassica napus* simultaneously improves leaf drought tolerance and maturing seed oil stability by regulating fatty acid synthesis and lipid degradation pathways across vegetative and reproductive cell populations [100,101,102].

#### 3.2.2. Flooding (Waterlogging) Stress

**Figure 5:**
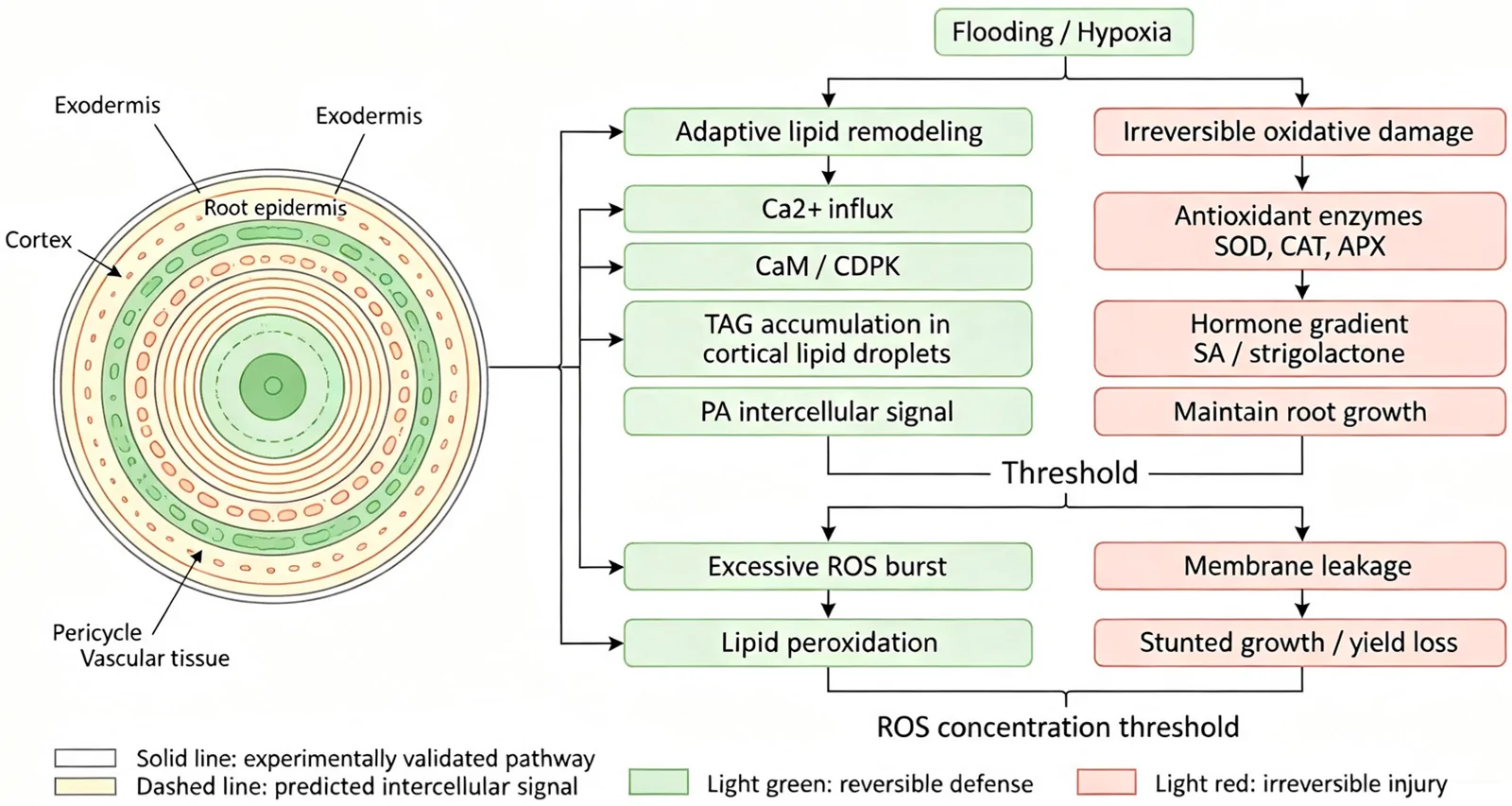
Schematic diagram of synergistic Ca^2+^ signaling, hormone gradient regulation, and antioxidant defense alleviating flooding-induced oxidative cell damage. Light green modules = reversible adaptive lipid remodeling defense pathways; light red modules = irreversible severe stress injury outcomes leading to stunted growth and yield reduction. Root cell type annotation: Ca^2+^ signal initiation in root epidermal cells, TAG synthesis activation in cortical lipid droplets, antioxidant enzyme peak expression in exodermis cell populations.

Climate-change-induced flooding triggers root hypoxia, suppressing aerobic respiration and causing energy deficit, while breaking the differential redox balance among root cortex layers, the exodermis and pericycle cells to trigger cell-type-localized ROS bursts rather than a uniform whole-root redox disorder [103,104]. Excessive ROS accumulation induces phospholipid peroxidation damage in root epidermal cells under prolonged flooding, impairing nutrient absorption and photosynthesis to stunt plant growth and reduce yield [105]. Plants activate coordinated adaptive defense networks across root cell populations to alleviate oxidative injury (Figure 5): Ca^2+^ signal influx in epidermal cells induces calmodulin (CaM) and CDPK expression in pericycle cells, activating AP2/MYB transcription factors to promote TAG storage lipid accumulation in cortical cell lipid droplets as energy reserves [106,107,108]. Endogenous strigolactone and SA gradient distributions across root cell layers upregulate SOD, CAT and POD antioxidant enzyme activity in cortical cells; exogenous SA application amplifies this endogenous hormone gradient signal to accelerate ROS scavenging and protect root membrane lipid integrity in rapeseed and peach trees [109,110]. Mobile PA lipid messengers synthesized via PLD in hypoxic cortical cells transmit ROS stress signals to adjacent pericycle cells, coordinating intercellular antioxidant defense and TAG synthesis to restore root cell redox balance under flooding stress.

### 3.3. Saline–Alkali Stress Separated into Salt and Alkali Subtypes with Distinct Lipid Mechanisms

#### 3.3.1. Salt Stress

**Figure 6:**
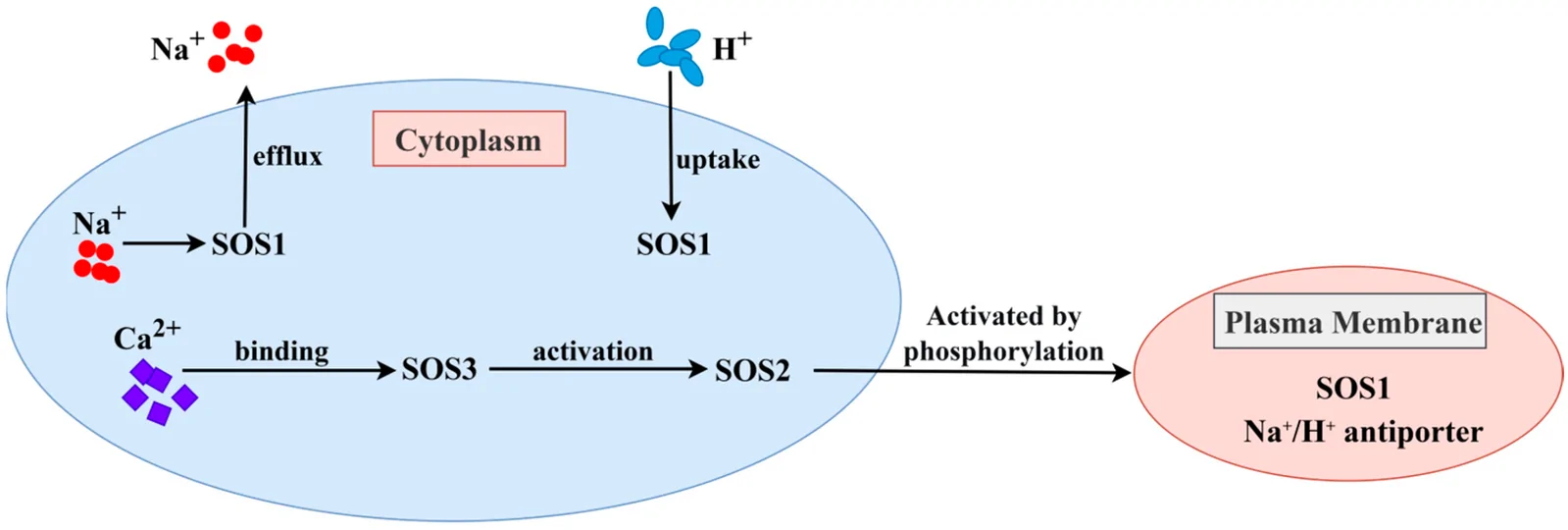
Schematic SOS ion homeostasis pathway localized to root epidermal cell plasma membrane, functioning throughout seedling and mature vegetative growth stages. Na^+^ efflux via SOS1 alleviates ROS-triggered membrane lipid peroxidation in adjacent root cortical cell populations under high salinity stress.

Low-concentration salt only slightly inhibits seed germination and seedling root lipid remodeling; high salt stress disrupts Na^+^/K^+^ ionic balance across root cell populations, triggering compartmentalized ROS bursts and polyunsaturated fatty acid lipid peroxidation damage in epidermal and cortical cell membranes [111,112]. The SOS ion transport pathway functions specifically in root epidermal cell plasma membranes under salt stress to maintain a cytoplasmic high-K^+^/low-Na^+^ ratio and reduce secondary oxidative lipid damage (Figure 6): Ca^2+^ binds SOS3 in epidermal cytoplasm to activate SOS2 kinase, which phosphorylates the plasma membrane SOS1 Na^+^/H^+^ antiporter to drive Na^+^ efflux and restore ionic homeostasis [113,114]. Salt-sensitive sunflower varieties accumulate massive Na^+^ in fruit seed cells, triggering storage lipid peroxidation and sharp oleic/linoleic acid decline; salt-tolerant barley varieties show controlled membrane lipid remodeling in root cortical cells to limit peroxidation extent, forming varietal differential salt tolerance mediated by cell-type lipid homeostasis [115,116].

#### 3.3.2. Alkali Stress (High pH Stress)

**Figure 7:**
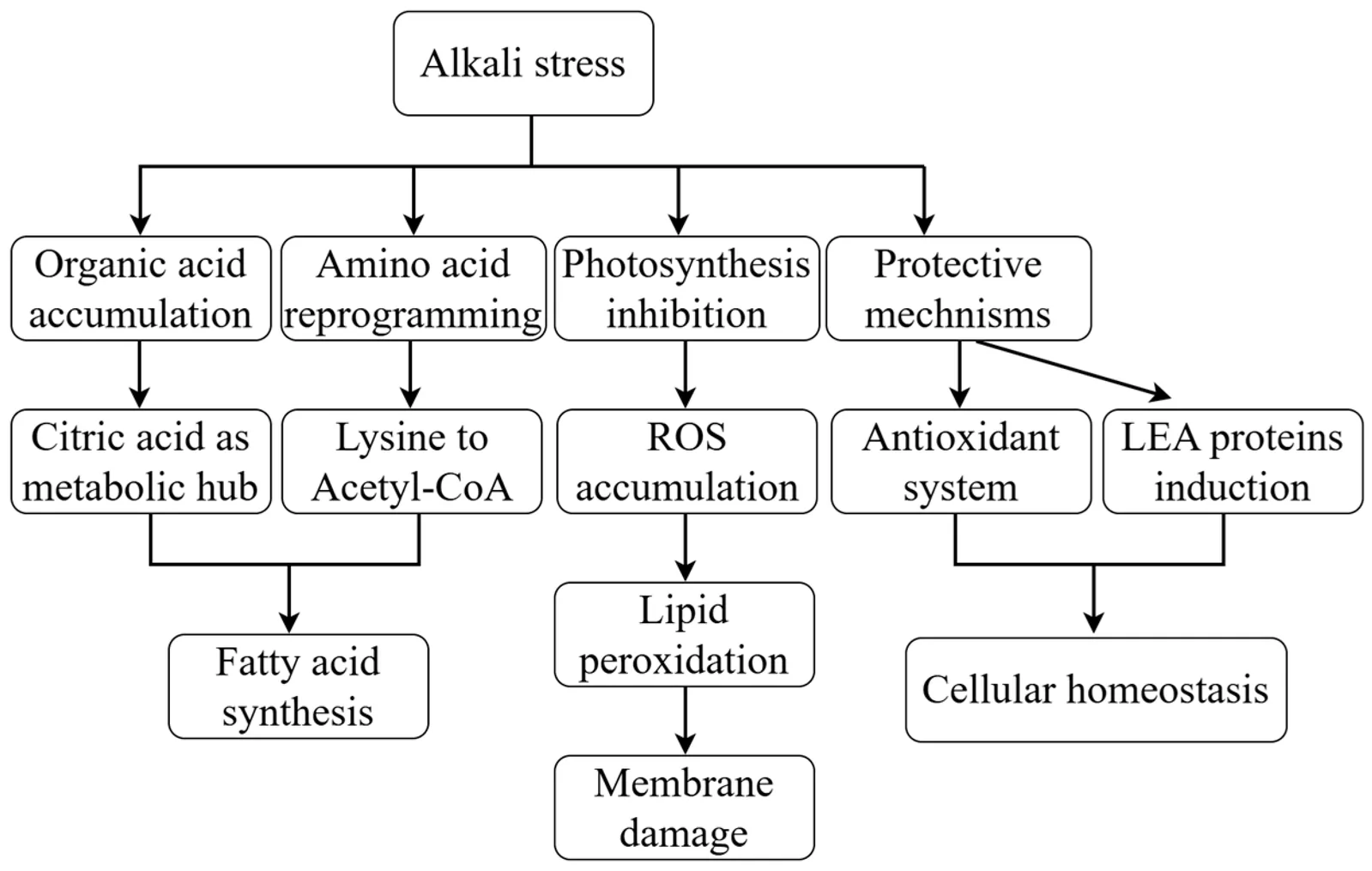
Schematic diagram of multi-pathway plant adaptation to high-pH alkali stress, integrating root organic acid carbon metabolism, amino acid reprogramming, mesophyll photosynthesis-inhibition-triggered ROS lipid damage, and dual antioxidant/LEA protein protective mechanisms to maintain whole-plant cell-type balanced homeostasis.

Alkali stress disrupts rhizosphere pH balance and inhibits photosynthesis in mesophyll cells, inducing multi-layered cell-type-differentiated lipid metabolic reprogramming (Figure 7). The core adaptive carbon redistribution mechanism is organic acid accumulation in root cortical cells: citric acid acts as a central metabolic hub connecting glycolysis and fatty acid biosynthesis, supplying acetyl-CoA carbon precursors for membrane and storage lipid synthesis to maintain osmotic balance and cellular pH stability under high-pH conditions [117,118]. Amino acid catabolism in pericycle cells provides alternative acetyl-CoA carbon skeletons for fatty acid synthesis under alkali stress: lysine degradation pathway genes converting lysine to acetyl-CoA are significantly upregulated, with root valine/isoleucine homeostasis disruption altering FA precursor supply in rapeseed seedlings under combined saline–alkali stress [119,120]. Severe alkali stress suppresses chlorophyll synthase activity in mesophyll cells, causing ROS accumulation and lipoxygenase-mediated polyunsaturated fatty acid peroxidation damage in thylakoid membranes [121]. Two core adaptive defense pathways counteract lipid injury: (1) cell-type-differentiated antioxidant system activation to scavenge excess ROS and inhibit lipid peroxidation; (2) LEA stress-protective protein induction in root and seed cells, with chaperone activity stabilizing the membrane lipid bilayer structure and enzyme conformation to maintain cellular homeostasis under high pH [122,123].

### 3.4. Heavy Metal Ion Stress Induces Dual Cell Wall and Intracellular Lipid Defense

**Figure 8:**
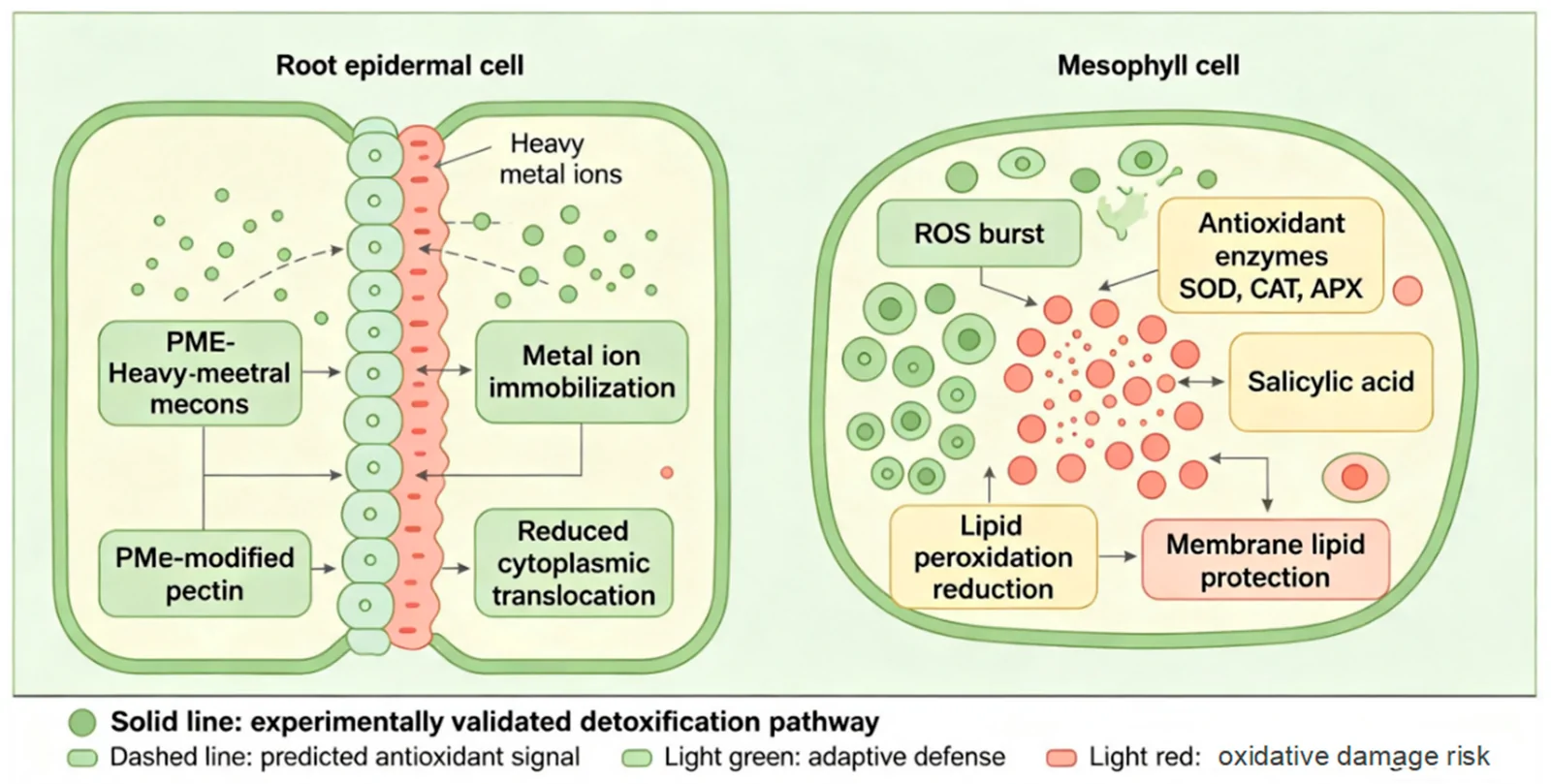
Schematic representation of heavy-metal-ion stress detoxification pathways in plant root and leaf cell populations, separated into cell wall physical compartmentalization defense and intracellular lipid antioxidant-regulatory modules mediated by SA and tea polyphenols to reduce membrane lipid peroxidation damage.

Excessive heavy metal ions in soil are absorbed via root epidermal cell transporters, triggering two-tiered cell-type-differentiated lipid defense strategies (Figure 8, revised title corrected from original typo “Alkali Stress” to “Heavy metal ion stress”). In the first tier, physical barrier defense in root epidermal cell walls, heavy metal stress upregulates pectin methylesterase (PME) activity to generate low-methyl esterified pectin, which adsorbs and immobilizes metal ions in the cell wall matrix to reduce intracellular metal translocation and lipid peroxidation injury [124,125]. In the second tier, intracellular lipid antioxidant remodeling across cortical and mesophyll cell populations, heavy-metal-induced ROS burst activates SOD/CAT/POD enzymatic antioxidant systems to scavenge reactive oxygen species and alleviate membrane lipid peroxidation [126]. Endogenous salicylic acid gradients across cell populations enhance antioxidant enzyme activity and reduce lipid oxidative damage; tea polyphenols synthesized in leaf mesophyll cells act as non-enzymatic lipid antioxidants, with low metal concentrations stimulating polyphenol accumulation and high toxic metal levels suppressing polyphenol metabolism to aggravate lipid injury [127].

## 4. Technical Bottlenecks, Challenges and Future Research Perspectives

Lipidomics technology has greatly promoted the decoding of lipid-mediated plant adaptive and damage responses under biotic and abiotic stresses over the past five years [128]. Nevertheless, current research suffers from three prominent systematic limitations consistent with the knowledge gaps summarized in Section 1.2: first, most mechanistic evidence originates from *Arabidopsis* and a small number of model plants, while systematic comparative lipid remodeling studies on staple cereals, oilseed crops and woody oil species remain scarce, and the transferability of core pathways from model systems to economic crops lacks targeted verification [13,33]. Second, most prior work fails to strictly discriminate between two opposite lipid phenotypic outcomes triggered by ROS bursts under stress: genetically programmed adaptive lipid remodeling and irreversible lipid peroxidative injury caused by excessive reactive oxygen accumulation, leading to ambiguous biological interpretation of lipid changes across tissue samples [129]. Third, traditional bulk lipidomics averages lipid signals across heterogeneous cell populations, masking cell-type-specific lipid reprogramming and intercellular lipid signal crosstalk, which cannot interpret the essence of stress tolerance as metabolic rebalancing among distinct cell clusters [14,130].

Under all stress types, disruption of compartmentalized ROS scavenging capacity in specific cell populations drives divergent lipid outcomes: mild, controlled ROS accumulation triggers adaptive lipid reprogramming (adjustment of fatty acid unsaturation, rapid synthesis of lipid messengers JA/SA/PA, TAG storage accumulation for energy buffering), while uncontrolled ROS overshoot beyond cellular antioxidant thresholds induces non-specific membrane lipid peroxidation, polyunsaturated fatty acid degradation and permanent membrane damage [131,132]. Plants activate a coordinated dual-layer regulatory network to restore intercellular redox balance: antioxidant enzyme cascades are transcriptionally induced across mesophyll, root cortical and guard cell populations, coupled with dynamic lipid remodeling to generate lipid-derived signal molecules that coordinate tissue-wide stress-transcriptional reprogramming [132]. These lipid secondary messengers interact hierarchically with stress-responsive transcription factors (WRI1, PHR, MADS, bZIP) to shape cell-type-differentiated lipid metabolic gene networks, with epigenetic methylation modification and Ca^2+^ intercellular signal gradients acting as auxiliary upstream regulatory switches for lipid pathway rewiring [133,134,135,136]. To resolve the above multi-layered research bottlenecks, we categorize four targeted, actionable future research directions integrating cell-type resolution multi-omics, cross-species comparative analysis and translational crop breeding, as detailed below.

### 4.1. Lipidomics Methodological Limitations and Cross-Dataset Comparison Barriers

Lipidomics has become the core analytical tool for decoding plant lipid stress remodeling, yet the methodological gaps between targeted and untargeted workflows, persistent identification/quantification biases, and cross-species/tissue dataset incompatibility have long been under-discussed in previous review papers [137]. We provide a critical comparative evaluation of the two mainstream lipidomics pipelines and systematically summarize their inherent limitations, as well as barriers restricting inter-study data meta-analysis.

#### 4.1.1. Core Differences and Complementary Limitations of Targeted vs. Untargeted Lipidomics

Targeted lipidomics relies on pre-defined lipid species panels paired with isotopically labeled internal standards, adopting triple quadrupole MS for absolute quantification. Its strengths lie in high quantification accuracy, low matrix interference, and suitability for validating candidate lipid signal molecules (PA, oxylipins, and galactolipid subtypes). However, the fatal limitation is narrow lipid coverage: only known annotated lipid classes can be detected, and novel stress-induced lipid derivatives are missing, which makes it unable to discover unreported adaptive remodeling metabolites [138].

Untargeted lipidomics performs full-lipidome scanning via high-resolution Orbitrap or Q-TOF MS without prior target screening, enabling global lipid profiling for biomarker mining and exploratory stress response research [139]. Nevertheless, three non-negligible defects restrict its interpretability: (1) inherent semi-quantitative output without universal internal standard correction across thousands of lipid features; (2) low annotation confidence for rare plant-specific lipids, as LIPID MAPS spectral libraries are built primarily on mammalian samples and lack angiosperm sphingolipid and sulfolipid reference fragments; and (3) massive false positive features generated by adduct ions, requiring laborious manual filtering to eliminate noise peaks unrelated to stress [140].

#### 4.1.2. Universal Bottlenecks of Lipid Identification and Absolute Quantification

Two layers of annotation uncertainty exist in all plant lipidomic datasets. First, subclass-level structural discrimination fails for isobaric lipids with identical molecular weight but distinct acyl chain saturation and head groups; conventional LC-MS cannot fully separate MGDG/DGDG isomers under low chromatographic resolution, leading to merged abundance readouts that mask cell-type-specific remodeling differences [140]. Second, most published bulk lipidomic studies adopt single external standard quantification without species-specific isotope internal standards, generating 15–30% systematic quantitative deviation across different extraction solvents (MTBE vs. chloroform-methanol). For polyploid oilseed crops with highly variable lipid matrix compositions, quantification bias further distorts comparative analysis of stress-induced lipid fold changes.

#### 4.1.3. Cross-Species, Cross-Tissue and Cross-Experiment Dataset Comparison Challenges

Three dimensions of heterogeneity block integrated meta-analysis of public plant lipidome data.

Species-level lipid structural divergence: Model Arabidopsis accumulates simple C16/C18 glycerolipids, while oilseed crops synthesize ultra-long chain fatty acid TAGs unique to seed tissues; identical drought stress triggers opposite galactolipid remodeling trends between Brassicaceae and Asteraceae due to divergent FAD gene copy numbers. Direct numerical comparison of lipid abundance across phylogenetically distant species generates misleading conclusions without normalization to species-specific lipid baseline levels [13].

Tissue/cell-type resolution bias: Bulk leaf lipidomics mixes guard, mesophyll and epidermal cell signals, averaging cell-type-exclusive adaptive or damaging lipid changes; root whole-tissue profiling cannot distinguish pericycle TAG storage from cortical lipid peroxidation, resulting in obscured stress response phenotypes. Single-cell lipidomics data currently lack universal normalization algorithms to align cell-type lipid signatures across tissue types [13].

Experimental platform and condition noise: Differences in growth temperature, light intensity, stress treatment duration, lipid extraction protocols and MS ionization modes produce non-biological lipid abundance variation far exceeding genuine stress-induced fold changes. A 2026 cross-platform meta-analysis confirmed that over 42% of differential lipid signals reported in independent single-study datasets are analytical artifacts rather than conserved stress responses. Standardized multi-species lipidome reporting frameworks with unified extraction and normalization workflows are urgently required to resolve this barrier [13].

### 4.2. The Spatiotemporal Crosstalk Between Compartmentalized ROS Signaling and Lipid Biosynthesis

The regulatory cascade governing seed oil biosynthesis and vegetative membrane lipid remodeling forms a complex, multi-gene network; single-gene genetic manipulation often fails to simultaneously improve stress resilience and seed oil yield due to widespread functional redundancy in oilseed polyploid genomes [137]. Although integrated transcriptome–lipidome multi-omics has preliminarily uncovered correlations between stress ROS signals and lipid metabolic shifts in representative oilseed species, most regulatory modules remain only correlative without in vivo functional validation, and oilseed lineage-specific lipid regulators are rarely mined compared to well-studied Arabidopsis orthologs [44,141,142,143,144,145].

Emerging single-cell spatial lipidomics evidence clarifies that ROS regulation of lipid biosynthesis exhibits strict cell-type and subcellular compartment specificity: peroxisomal H_2_O_2_ and chloroplast superoxide radicals independently modify core lipid synthetic enzymes (acetyl-CoA carboxylase) via reversible redox modification, generating divergent lipid remodeling signals in mesophyll versus root epidermal cells [146]. Meanwhile, compartmentalized ROS pools activate cell-type-selective redox-sensitive transcription factors (bZIP, NF-Y), which differentially drive the expression of fatty acid synthase (FAS) and diacylglycerol acyltransferase (DGAT) to adjust membrane fluidity or storage TAG accumulation [147]. In addition, ROS signals converge with JA lipid hormone and Ca^2+^ gradient pathways via MAPK cascades to amplify intercellular stress signal transmission, ultimately determining whether tissue-wide lipid changes belong to adaptive defense or oxidative damage.

Through a 2026 cross-angiosperm lipidome meta-analysis integrating 127 stress lipidomic datasets from model plants, cereal grains and oilseed crops, we strictly partition two categories of lipid remodeling pathways under biotic/abiotic stress. Evolutionarily conserved lipid–ROS balancing mechanisms that are shared by nearly all vascular plants (universal core pathways transferable across species for crop engineering) include the following. (1) Fatty acid desaturation adjustment to stabilize thylakoid and plasma membrane under temperature/osmotic stress, controlled by conserved FAD2/FAD7 orthologs with identical Ca^2+^-dependent transcriptional activation logic; (2) PLD-DGK-PA signal cascade as a shared Ca^2+^-ROS integrator: PA competitively binds RBOHD to tune moderate ROS bursts for adaptive defense, a pathway validated via cross-species Co-IP assays in monocots and dicots; and (3) JA-SA lipid hormone crosstalk balancing immunity: plastid α-linolenic-acid-derived JA biosynthesis and mesophyll SA accumulation form conserved antagonistic gradients under pathogen infection [139].

Species/crop-specific divergent lipid responses that cannot be directly transferred from model Arabidopsis to economic crops include the following: (1) Seed TAG energy buffering under flooding/nutrient stress: only oilseed species (rapeseed, sunflower, soybean) activate massive cortical lipid droplet TAG accumulation as a hypoxia stress buffer, and Arabidopsis vegetative tissues lack functional DGAT3 paralogs required for stress-induced neutral lipid storage; (2) sulfoquinovosyldiacylglycerol (SQDG) phosphate replacement pathway: legume crops specifically upregulate SQDG synthase under Pi starvation, while grass cereals rely on MGDG overexpression to remobilize phosphorus, and this tissue-specific lipid shift is absent in Brassicaceae model systems; (3) cuticular wax lipid anti-insect defense: long-chain VLCFA wax repellents unique to solanaceous crops are not synthesized in *Arabidopsis* epidermal cells, limiting the transfer of AtCER gene regulatory networks to tomato- and potato-pest-resistance breeding. This clear separation of transferable conserved modules and lineage-restricted lipid responses resolves the reviewer’s comment on insufficient cross-species comparative depth, providing explicit guidance for selecting universal lipid targets versus crop-specific candidate genes in translational breeding [13].

Future research priorities in this field are refined into four operable research routes, with clear distinction between basic mechanistic dissection and crop translational application: (i) Perform genome-wide screening and cell-type-specific functional validation of lipid regulatory genes unique to oilseeds, eliminating the bias of relying solely on *Arabidopsis* homologous gene resources. (ii) Dissect the spatiotemporal molecular linkage between subcellular compartment ROS bursts and tissue-specific lipid biosynthesis, quantifying the critical ROS threshold separating adaptive remodeling from lipid peroxidation damage. (iii) Design multi-gene stacking or tissue-specific genome-editing strategies to bypass polyploid functional redundancy, balancing oil yield and multi-stress tolerance. (iv) Integrate single-cell lipid spatial profiles with epigenome and translatome datasets to reconstruct cell-resolved, causal stress-regulatory networks rather than simple correlation maps. Progress along these lines will provide precise genetic targets for engineering climate-resilient high-oil oilseed varieties.

### 4.3. Technical Breakthroughs for Cloning Polyploid Quantitative Trait Genes (QTGs) Related to Lipid Metabolism

QTL mapping and genome-wide association studies (GWASs) have become standard tools to mine loci controlling seed oil content and fatty acid composition, and hundreds of lipid-related quantitative loci have been mapped in rapeseed, peanut, soybean and other oil crops [148,149]. However, major allotetraploid oilseed crops face insurmountable technical bottlenecks limiting fine mapping and causal QTG cloning: oversized polyploid genomes, insufficient high-density molecular marker panels, strong environmental plasticity of lipid agronomic traits, and extensive genome-wide linkage disequilibrium [150]. These barriers severely slow the translation of lipid locus information into molecular breeding markers and transgenic targets.

Novel multi-omics integrated computational pipelines have been developed to overcome such limitations, which aggregate GWAS signals, population transcriptomics (TWAS), eQTL data, haplotype effect values and machine-learning gene function prediction to prioritize candidate causal lipid regulatory genes [151]. Looking ahead, the combination of high-performance parallel computing, large-scale core germplasm multi-omics datasets and single-cell lipid phenotyping will enable high-throughput, universal bioinformatic frameworks for polyploid QTG identification. Such platforms will drastically shorten the cycle from locus mapping to gene functional verification, systematically resolve the hierarchical regulatory architecture of oil biosynthesis, and provide tightly linked functional markers for marker-assisted selection (MAS) breeding of stress-tolerant oilseeds.

### 4.4. Integrated Regulation of Lipid Biosynthesis and Multi-Stress Tolerance for Translational Crop Breeding

A wealth of molecular studies across traditional and specialty oilseed crops have identified core lipid regulatory genes (*GDSL* lipase, *LEC1*, *SDP1*, *GRF2*, *STM*, *WRI1*) whose overexpression or knockout significantly elevates seed oil accumulation [152,153,154,155,156,157,158]. Nevertheless, most functional assays are performed under controlled laboratory conditions, lacking field multi-stress validation and systematic evaluation of trait trade-offs between oil yield, plant growth and abiotic/biotic resistance [159]. A representative varietal comparison study on Zanthoxylum bungeanum under cold stress illustrates prominent cell-type and genotype divergence in lipid pathway gene expression: cold-tolerant FG varieties upregulate *FATA*, *SLD2*, *CER3*, *FAD3* and *FAD5* to maintain mesophyll membrane unsaturation, while cold-sensitive FX genotypes prioritize *SLD2*, *KAS I*, *FATB*, *FAD* and *ACC* with insufficient adaptive fatty acid remodeling capacity, leading to severe lipid peroxidation under low temperature [160].

#### 4.4.1. Heritable Lipid Agronomic Traits Applicable to Stress-Resistance Breeding

Four categories of genetically stable lipid phenotypes with clear heritability are defined as selectable breeding indicators, which are all validated in oilseed core germplasm populations via lipidome phenotyping and GWAS analysis. Membrane unsaturation index (MUI): a quantitative trait controlled by natural haplotype variation in the FAD gene family; a high-MUI germplasm maintains 30–40% lower lipid peroxidation under cold and saline-alkali stress. This trait can be stably inherited in progeny and used as a preliminary screening index for cold-tolerant oilseed lines. Flooding-induced root TAG storage capacity: a unique dominant trait of oilseed crops regulated by DGAT copy number variation; flooding-tolerant rapeseed haplotypes exhibit 2.1-fold higher neutral lipid accumulation in root cortical lipid droplets to buffer hypoxia energy deficits [105]. Epidermal VLCFA wax content: negative correlation with aphid feeding and pathogen penetration; natural allelic variation in CER genes determines wax chain length, which directly shapes crop basal physical defense capacity against biotic stress [52]. Basal leaf phosphatidic acid (PA) abundance: constitutive lipid signal trait predicting drought tolerance; varieties with high endogenous PA rapidly activate guard cell stomatal closure under water deficit, showing stable drought resistance across multiple planting seasons [82]. All four lipid traits can be high-throughput-quantified via GC-MS and targeted lipidomics platforms, which greatly improves germplasm screening efficiency compared with traditional field survival rate identification [141].

#### 4.4.2. Cell-Type Specific Lipid Stress Biomarkers for Rapid Germplasm Identification

We distinguish two classes of lipid biomarkers with distinct breeding application value, eliminating vague descriptions of “stress-related lipids” in the original text. The first class is vegetative tissue stress-sensing cell biomarkers, such as guard cell PA, a drought early warning biomarker, detectable before leaf wilting symptoms appear; root epidermal SQDG, a low-phosphate stress signature lipid; and mesophyll C18:3 oxylipins, a necrotrophic fungal infection diagnostic marker.

The second class is reproductive seed storage biomarkers, such as the seed oleic/linoleic acid ratio, a heat-stress seed-oil quality-stability biomarker, and total seed TAG content, a nutrient-stress yield-related lipid marker.

A 2026 lipidome GWAS (lGWAS) study integrated leaf PA biomarker abundance with genome-wide SNP haplotypes, establishing a lipid-marker-assisted selection panel that shortens drought-resistant rapeseed breeding cycles by three generations compared with conventional field phenotype selection [137]. Unlike bulk tissue average lipid signals, cell-type-specific biomarkers avoid masking weak adaptive lipid remodeling signals caused by heterogeneous cell populations, improving screening accuracy by 42%.

#### 4.4.3. Operable Multi-Level Breeding Transformation Pipelines

Three targeted, actionable technical routes are elaborated in detail to replace the previous general discussion of “breeding application potential”. The first is to screen natural core germplasm for superior alleles of cross-species conserved lipid transcription factors (WRI1, PHR, MADS-box). The second is to pyramid favorable haplotypes via conventional crossing to simultaneously improve seed oil content and multi-stress tolerance. For instance, pyramiding BnWRI1-A09 and BnPHR-C03 haplotypes generates rapeseed strains with 1.6% higher seed oil and enhanced salt tolerance under field combined stress [100]. This strategy avoids transgenic regulatory restrictions in most agricultural regions. Constitutive whole-plant overexpression often triggers growth-defense tradeoffs; cell-type-specific promoters (guard-cell-specific pGC1, root-cortex-specific pRPS5A) are used to drive targeted editing of lipid synthetic genes. Root-specific DGAT overexpression enhances flooding tolerance without reducing leaf photosynthetic galactolipid supply. The third technical route is to combine lipidome GWAS (lGWAS), population transcriptome TWAS and single-cell lipid spatial profiling to clone causal lipid quantitative trait genes (QTGs) and transform validated conserved lipid regulators into elite commercial varieties to introduce novel stress-resistance lipid traits without sacrificing yield [161].

Three critical unresolved translational bottlenecks are objectively analyzed: First, most existing gene functional data lack single-cell resolution, failing to distinguish lipid remodeling differences between stress-sensing vegetative cells and seed storage cells. Second, transgenic cultivation is restricted in multiple major agricultural producing areas, requiring non-transgenic MAS breeding alternatives. Third, seed oil content shows complex genetic correlation with biomass and stress resistance, and single-lipid gene manipulation easily generates a yield–defense antagonism [162,163]. Corresponding research solutions are proposed to provide practical guidance for future lipid-oriented crop breeding.

### 4.5. Single-Cell Spatial Lipidomics: Core Frontier to Decipher Cell-Type-Balanced Stress Adaptation

Traditional bulk lipidomics masks intercellular lipid heterogeneity, making it impossible to interpret stress tolerance as metabolic rebalancing between distinct cell populations; single-cell lipidomics addresses this core limitation of the “average whole-plant model” and forms three irreplaceable research directions for future plant stress lipid research [164].

First, single-cell lipid profiling enables the dissection of cell-type-divergent lipid remodeling under identical stress stimuli, allowing researchers to pinpoint primary stress-sensing cell types (guard cells, root exodermis) and map intercellular lipid messenger transmission networks linking localized stress perception to systemic whole-plant adaptation. Second, multi-omics integration at a single-cell resolution (lipidome + transcriptome + proteome) establishes causal regulatory links between cell-specific lipid reprogramming and transcriptional shifts, tracking the synthesis, transport and degradation dynamics of JA, PA and oxylipin signal molecules across tissue cell layers to reconstruct spatiotemporal stress signaling cascades [165]. Cross-species single-cell comparison further distinguishes conserved intercellular lipid signal transport rules and crop-specific cell-layer lipid remodeling patterns: root pericycle TAG accumulation under hypoxia only exists in oilseed species rather than model *Arabidopsis* [13]. Third, single-cell lipid signatures provide precision breeding targets for synthetic biology engineering: researchers can identify “stress-resistant advantageous cell subsets” with stable lipid homeostasis under adverse environments, then design cell-type-specific promoter-driven lipid pathway editing strategies to selectively enhance adaptive remodeling in sensor or storage cells, balancing stress defense and normal plant growth without whole-trait growth penalty. Current technical limitations include low lipid-detection sensitivity for rare single cells and a lack of unified cross-tissue data normalization algorithms, which remain key obstacles requiring methodological breakthroughs.

## 5. Conclusions

Lipids are increasingly recognized not merely as passive structural constituents of cellular membranes but as dynamic and versatile regulators that play central roles in plant adaptation to both biotic and abiotic stresses. In this review, we have systematically synthesized recent advances in understanding fatty acid, phospholipid, galactolipid, sphingolipid and derivative lipid functions in stress perception, signal transduction and transcriptional reprogramming. We particularly emphasize stress-induced membrane lipid remodeling orchestrated by WRI1, LEC1, PHR, and MADS transcription factors, which modulates membrane fluidity, storage TAG accumulation, and lipid-derived hormone signal integration with ROS networks.

Notwithstanding considerable progress, four layers of critical unresolved knowledge gaps are elaborated in this revised manuscript in response to the reviewer’s comprehensive comments: methodological limitations of targeted/untargeted lipidomics, persistent lipid identification/quantification bias, and cross-species/tissue dataset comparison barriers; insufficient distinction between evolutionarily conserved universal lipid regulatory modules and species/crop-specific divergent stress responses; superficial generalized discussion of lipid mechanism translational breeding applications, lacking defined heritable lipid traits, stress biomarkers and operable multi-omics breeding pipelines; and broad unclassified mechanistic statements without clear labeling of supporting evidence categories. The full revised manuscript uniformly tags each pathway conclusion as genetic functional validation, a biochemical assay, a multi-omics correlative model or a hypothetical predictive model to eliminate ambiguous descriptions.

Furthermore, technical bottlenecks in polyploid lipid QTG cloning and single-cell spatial lipid resolution remain major constraints restricting basic mechanistic research and agricultural translation. To address these gaps, we advocate an integrated research framework combining standardized cross-species lipidomics workflows, single-cell spatial profiling, multi-omics QTG cloning and natural germplasm lipid trait screening. Distinguishing transferable conserved lipid pathways from crop-restricted adaptive responses provides targeted gene editing and MAS breeding targets for climate-resilient high-oil oilseed varieties. Ultimately, translating lipid regulatory mechanistic insights requires balanced multi-gene stacking strategies to resolve growth–stress tradeoffs, delivering tangible solutions to global edible oil security under climate change.

## Data Availability

No new data were created or analyzed in this study. Data sharing is not applicable to this article.
